# Accuracy of Visual Estimation of Active Shoulder Elevation Among Orthopedic Physicians: An Exploratory Video-Based Study

**DOI:** 10.7759/cureus.116206

**Published:** 2026-09-14

**Authors:** João Artur Bonadiman, Guilherme Vilela, José Elías León Donis, Carlos Rodriguez, Gustavo Villalba, Paulo Piluski, Osvandre Lech

**Affiliations:** 1 Orthopaedics, Hospital São Vicente de Paulo, Instituto de Ortopedia e Traumatologia, Passo Fundo, BRA; 2 Orthopaedics, Instituto Brasil de Tecnologias da Saúde, Rio de Janeiro, BRA; 3 Orthopaedics, Centro Hospitalario La Paz, Ciudad de Guatemala, GTM; 4 Orthopaedics, Clinic Trauma, Ciudad del Este, PRY

**Keywords:** measurement accuracy, orthopedic education, range of motion, shoulder joint, telemedicine, visual estimation

## Abstract

Introduction

Visual estimation of shoulder range of motion (ROM) is rapid and widely used, but its accuracy may vary across the arc of elevation. This study quantified angle-specific error in the visual estimation of active shoulder elevation by orthopedic physicians.

Materials and methods

In this cross-sectional video-based study, 50 physicians at different stages of orthopedic training viewed seven approximately 10-second videos showing active shoulder elevation at six reference angles: 30° (recorded twice, once in the coronal plane and once in the sagittal plane), 60°, 70°, 90°, 115°, and 160°. The reference angles were measured with a universal goniometer and checked with the phyphox smartphone application. Participants used a slider ranging from 0° to 180° to record each estimate. The primary outcome was signed estimation error, calculated as the estimated angle minus the reference angle. Exact agreement was analyzed descriptively. Angle-specific one-sample t-tests assessed whether the mean signed error differed from zero, with Holm correction for seven comparisons. Mean absolute error per participant was compared across training levels.

Results

The 50 participants generated 350 ratings. Mean estimates were closest to the reference value at 90° and 115°, with a mean signed error of -0.7° for both angles. After Holm correction, the only angle showing a statistically significant bias was 160°, which was underestimated by a mean of 21.4° (95% CI, -25.7° to -17.2°; adjusted p < 0.001). The 70° target was overestimated by 5.2° (95% CI, +1.3° to +9.2°; adjusted p = 0.063), and the 30° target recorded in the sagittal plane was overestimated by 5.8° (95% CI, -0.5° to +12.2°; adjusted p = 0.361). Neither comparison remained statistically significant after Holm adjustment for seven angle-specific tests. Exact agreement was highest at 90° (43/50, 86%), followed by 30° in the sagittal plane (23/50, 46%) and 30° in the coronal plane (19/50, 38%). Mean absolute error did not increase with less experience: it was lowest among third-year residents and did not differ between residents, fellows, and specialists as groups.

Conclusion

In this video-based setting, visual estimation of active shoulder elevation was not uniformly accurate and showed substantial angle-dependent bias, particularly marked underestimation at high elevation. Visual estimation may provide a rapid gross impression of shoulder elevation; however, standardized goniometric or validated digital measurement should be preferred when precise documentation or detection of change is required.

## Introduction

Range of motion (ROM) is a central component of the shoulder examination and is incorporated into clinical decision-making, postoperative follow-up, and commonly used outcome scores, such as the American Shoulder and Elbow Surgeons (ASES) score [1] and the Constant-Murley score [2], which guide numerous scientific studies. The validity of clinical comparisons depends on measurements that are sufficiently accurate and reproducible. Universal goniometry is widely used and can provide acceptable reliability when the examination technique is standardized [3-5]. These two properties should not be conflated. Reproducibility describes the extent to which repeated assessments agree, whether by the same or different observers, whereas validity requires comparison against an appropriate reference standard. Observers may therefore agree closely with one another while sharing the same systematic error, so reproducibility alone does not establish accuracy. Nevertheless, visual estimation remains common because it is rapid, requires no equipment, and can be performed during routine office visits or remote consultations.

Visual estimation of ROM is not confined to orthopedic surgeons. Patients with shoulder complaints present to physicians, physiotherapists, occupational therapists, and chiropractors, and shoulder ROM is documented visually across these settings [6,7]. Visual estimates recorded by any of these professionals may be entered into clinical records and outcome instruments, so the accuracy of visual estimation is relevant across musculoskeletal care rather than to orthopedic practice alone.

Prior studies have shown that the reliability of shoulder ROM measurement varies according to the movement assessed, examiner, protocol, and instrument [8-10]. Visual estimation may produce broad measurement error. Alternative instruments have been studied separately and do not carry equivalent evidence. Accelerometer-based smartphone inclinometry has shown acceptable to good validity against goniometry for shoulder elevation in the sagittal and coronal planes [10], digital inclinometers have shown reproducibility comparable to or better than universal goniometry in patients with shoulder pain [5], and motion-analysis and pose-estimation applications remain under evaluation [11,12]. Reported performance varies with the plane of motion, the device and application, the population studied, and the measurement protocol, and findings from one combination should not be generalized to others. The expansion of telemedicine has further increased the practical relevance of remote visual assessment and has highlighted the need to understand its limitations [13].

Recent work involving shoulder surgeons found that remote visual estimates can be reasonably consistent between observers while still showing limited accuracy relative to digital reference measurements [14]. This distinction is important: observers may agree with one another yet share the same systematic bias. Angle-specific error may also be clinically meaningful because recognizable reference positions, such as 90° of elevation, may be easier to estimate than intermediate or terminal positions.

The primary objective of this exploratory study was to quantify the angle-specific accuracy of visual estimation of active shoulder elevation by orthopedic physicians, expressed as the mean signed error for each of seven angle-plane conditions and analyzed as a single family of seven comparisons. Secondary objectives were descriptive and comprised the dispersion of estimates and the frequency of exact agreement at each angle. A comparison between the coronal and sagittal planes at the single reference angle recorded in both planes (30°) was exploratory and post hoc; the study was not designed to compare planes. We hypothesized that estimation error would not be uniform throughout the elevation arc and that some reference angles would demonstrate systematic overestimation or underestimation.

## Materials and methods

Study design and ethics

This was a cross-sectional, video-based measurement study involving orthopedic residents, shoulder and elbow fellows, and orthopedic surgeons specializing in shoulder and elbow surgery. The study protocol was initially submitted in 2022 to the Research Ethics Committee of the University of Passo Fundo (CEP-UPF; CAAE 62959322.2.0000.5342; issued 28 September 2022, before recruitment began) and was subsequently updated, following a change in the corresponding author’s institutional affiliation, to the Research Ethics Committee of the Federal University of Fronteira Sul (CEP/UFFS; approval no. 7,884,061; CAAE 89431725.0.0000.5564). Electronic informed consent was obtained from all participating physicians: the opening screen of the online questionnaire presented the full informed consent document approved by the committee, and participants recorded their agreement by selecting “Accept” before any video was displayed (Figure 1). No paper consent forms were collected, and no names or contact details were requested at any stage.

**Figure 1:**
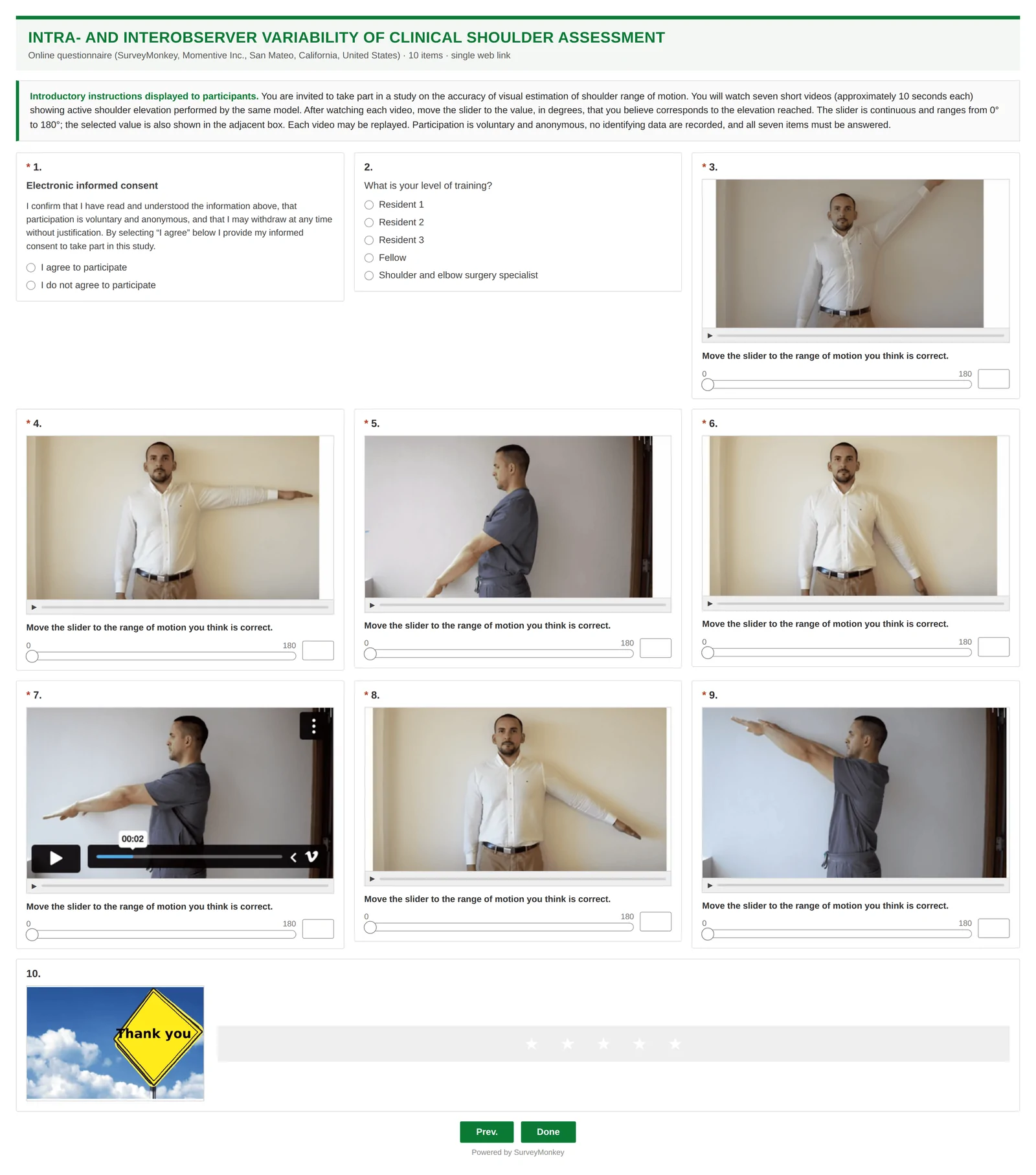
Complete study questionnaire Complete version of the online questionnaire (SurveyMonkey; Momentive Inc., San Mateo, CA, USA) developed by the authors for this study and reproduced in a panel layout for legibility. The instrument comprised 10 items: the informed consent document displayed on the opening screen, which participants accepted electronically before any video was shown (item 1; reproduced in the original Portuguese, with an English translation added for the reader); the participant’s level of training, with its five response options (item 2); the seven video-based assessments of active shoulder elevation (items 3-9), each answered on a continuous slider ranging from 0° to 180°, with the selected value also displayed in the adjacent numeric box; and the closing screen (item 10). Items 3-9 correspond to Videos 1-7, respectively. The images shown are the opening still frames of the embedded videos. Only the page layout was changed; no item wording was altered. Written and signed informed consent was obtained for publication of the identifiable facial image in an open-access journal.

Video preparation and reference measurements

All videos featured the same asymptomatic male model, assessed in the standing position; the left shoulder was evaluated in all seven videos. Seven short videos, each approximately 10 seconds in duration, demonstrated active shoulder elevation from the neutral position to a predefined endpoint. Prior to filming, each target position was measured with a 12-inch transparent universal goniometer (Baseline®, Fabrication Enterprises Inc., Elmsford, NY, USA) following the standard technique and landmarks described by Norkin and White [15], with the axis centered over the lateral aspect of the acromion process, the stationary arm aligned with the lateral midline of the thorax, and the moving arm aligned with the lateral midline of the humerus and directed toward the lateral epicondyle. Four videos were recorded in the coronal (frontal) plane and three in the sagittal plane; the 30° position was recorded in both planes (160°, Video 1, coronal; 90°, Video 2, coronal; 30°, Video 3, sagittal; 30°, Video 4, coronal; 70°, Video 5, sagittal; 60°, Video 6, coronal; 115°, Video 7, sagittal). The model reproduced the position, which was re-measured and adjusted iteratively until the goniometric reading matched the intended reference angle.

**Video 1:** Active shoulder elevation to 160° in the coronal plane Written and signed informed consent was obtained for publication of the identifiable facial image in an open-access journal.

**Video 2:** Active shoulder elevation to 90° in the coronal plane Written and signed informed consent was obtained for publication of the identifiable facial image in an open-access journal.

**Video 3:** Active shoulder elevation to 30° in the sagittal plane Written and signed informed consent was obtained for publication of the identifiable facial image in an open-access journal.

**Video 4:** Active shoulder elevation to 30° in the coronal plane Written and signed informed consent was obtained for publication of the identifiable facial image in an open-access journal.

**Video 5:** Active shoulder elevation to 70° in the sagittal plane Written and signed informed consent was obtained for publication of the identifiable facial image in an open-access journal.

**Video 6:** Active shoulder elevation to 60° in the coronal plane Written and signed informed consent was obtained for publication of the identifiable facial image in an open-access journal.

**Video 7:** Active shoulder elevation to 115° in the sagittal plane Written and signed informed consent was obtained for publication of the identifiable facial image in an open-access journal.

Each position was additionally verified with the phyphox application (version 1.1.11; RWTH Aachen University, Aachen, Germany) installed on an Apple iPhone running iOS 16 (Apple, Inc., Cupertino, CA, USA), using the Inclination module with the device secured to the lateral aspect of the distal arm, just proximal to the lateral epicondyle. The Inclination module reports device tilt derived from the internal accelerometer relative to the gravitational vertical. Phyphox is a general-purpose sensor-acquisition platform and, to our knowledge, has not been formally validated as a clinical goniometric instrument for shoulder ROM; it was therefore used as a secondary internal consistency check on the goniometric reference positions rather than as a criterion standard. Accelerometer-based smartphone inclinometry more broadly has shown acceptable to good validity and reliability for shoulder elevation [10,12], although performance is device- and protocol-dependent. Because phyphox measures inclination relative to the gravitational vertical, whereas goniometry references the trunk, the readings are directly comparable only when trunk compensation is minimal. The model was healthy, asymptomatic, and instructed to keep the trunk upright and still, and trunk position was monitored visually by the examiner during each recording; however, trunk position was not independently quantified by any instrument.

A position was accepted when the two methods agreed within 5°. This tolerance was defined a priori because it approximates the reported measurement error of universal goniometry at the shoulder [3,4] and corresponds to the threshold conventionally used to define precision in range-of-motion validation studies [16]. We acknowledge that a fixed 5° tolerance is proportionally larger at low target angles (17% of a 30° target) than at high ones (3% of a 160° target). Each position was checked against this criterion in real time and adjusted until the criterion was met; the individual paired readings were not recorded in writing at the time of filming and are therefore not available for tabulation. All measurements were verified by one of the co-authors (J.A.B.), a shoulder and elbow surgeon with five years of experience, immediately before each recording. Two goniometric measurements were obtained per position, one before and one after repositioning of the model. Videos were recorded with an iPhone 14 Pro (Apple Inc., Cupertino, CA, USA) running iOS 16.1 in landscape orientation, perpendicular to the plane of movement, positioned 3 m from the model and 1.50 m above the floor; camera position and distance were checked before each video. The recordings were edited using Adobe Premiere Pro 2022 (Adobe Inc., San Jose, CA, USA). The final videos were hosted on Vimeo (Vimeo Inc., New York, NY, USA) and embedded in an online SurveyMonkey questionnaire (Momentive Inc., San Mateo, CA, USA). The listed reference angles represent the recorded goniometric values used as the study reference positions rather than rounded target values.

Participants and survey procedure

All orthopedic residents (n = 23) and shoulder and elbow fellows (n = 8) of a single high-complexity referral center were invited by email through the training program, and the invitation was reinforced in person at departmental meetings. Shoulder and elbow specialists (n = 51) were invited individually by email only and were eligible if they had at least three years of practice in shoulder and elbow surgery. Participants were deliberately sampled across the full range of orthopedic training, from first-year residency to subspecialty practice, because visual estimation of shoulder elevation is performed and recorded by clinicians at every one of these levels; the intention was to reflect the conditions under which such estimates are made in routine care rather than to characterize the performance of a single homogeneous group. Recruitment followed a convenience sampling strategy. Of the 82 physicians invited, 50 completed the questionnaire between 23 November 2022 and 3 January 2023 (overall response rate, 61.0%); 14 further entries were opened but not completed and were excluded. Response rates differed markedly by subgroup: 23/23 (100%) among residents, 8/8 (100%) among fellows, and 19/51 (37%) among specialists. The overall rate therefore conceals differential participation by training level, and the specialist subgroup represents a self-selected minority of those invited.

Responses were collected prospectively and anonymously through a single web link. No names, email addresses, or other contact details were requested. The survey platform logged the IP address and the start and completion times of each submission by default; these data were used only for a post hoc audit of respondent uniqueness. IP addresses were retained in the original platform export but removed from the de-identified dataset provided as supplementary material. No device-level, network-level, or account-level restriction on repeat submission was applied. A post hoc audit of the 50 completed responses found no identical response sets; three IP addresses were shared by two or three completed responses submitted within minutes of each other from the institutional network, and in every case, the response sets differed on at least three of the seven angles, consistent with distinct respondents on a shared network. Uniqueness of respondents cannot, however, be verified with certainty. After viewing each video, the participant selected the perceived endpoint angle using a continuous slider ranging from 0° to 180°. Each physician rated all seven videos, yielding 350 angle estimates. The videos were presented to every participant in the same fixed order (Videos 1-7). The questionnaire was developed by the authors specifically for this study and is provided in full in Figure 1; no previously published or copyrighted instrument was used. The primary analysis was performed in July 2023, and the participant-level analyses described below were performed in September 2026.

Outcomes and statistical analysis

The primary scientific endpoint was the family of seven angle-specific mean signed errors, analyzed together with Holm control across the seven comparisons [17]. No individual angle was prespecified as the primary target; the prominence of the 160° result is therefore a finding of the study rather than a prespecified hypothesis. Signed estimation error for each target angle was defined as the estimated angle minus the reference angle. Negative values indicated underestimation, and positive values indicated overestimation. For each angle, the mean estimate, standard deviation, mean signed error, and 95% CI were reported. A two-sided one-sample t-test assessed whether the mean signed error differed from zero. Exact agreement, defined as an estimate identical to the reference angle, and the dispersion of estimates were secondary descriptive endpoints; because the response tool used a continuous slider, exact agreement was considered a secondary descriptive measure rather than the principal measure of accuracy.

All analyses were performed on the participant-level dataset exported from the survey platform (50 complete responses and 14 incomplete entries excluded). In addition to the angle-specific analyses, the following were performed: exact agreement and the proportion of estimates within ±5° and ±10° of the reference at each angle; mean absolute error per participant across the seven videos, compared across training levels with the Kruskal-Wallis test and pairwise Mann-Whitney tests with Holm correction; a linear mixed-effects model of absolute error with video and training group as fixed effects and a random intercept for participant; a paired comparison of the coronal and sagittal 30° videos (paired t-test and Wilcoxon signed-rank test); the intraclass correlation coefficient for absolute agreement among raters across the seven videos (ICC(2,1)); and an audit of shared IP addresses and completion times. These analyses were not prespecified and are interpreted as exploratory. Because all participants viewed the videos in the same fixed order, order effects cannot be separated from angle effects.

Descriptive statistics were generated in IBM SPSS Statistics for Windows, Version 27 (Released 2019; IBM Corp., Armonk, NY, USA); all other analyses were performed in Python, version 3.11 (Python Software Foundation, Wilmington, DE, USA), with SciPy 1.17 and statsmodels 0.15. Statistical significance was set at p < 0.05. No a priori sample-size calculation was performed because this was an exploratory study based on a convenience sample. Post hoc, with 50 raters, the half-width of the 95% CI for the mean signed error ranged from ±1.5° to ±6.4° across angles, providing adequate precision to identify systematic bias of clinically relevant magnitude (≥10°). The de-identified participant-level dataset is provided in Appendix 1.

## Results

Participant characteristics

All 50 participating physicians completed estimates for the seven target angles, resulting in 350 observations. The cohort included 19/50 (38%) shoulder and elbow specialists, 8/50 (16%) fellows, 8/50 (16%) third-year orthopedic residents, 8/50 (16%) second-year orthopedic residents, and 7/50 (14%) first-year orthopedic residents. The distribution by training level, together with the number invited and the response rate in each subgroup, is shown in Table 1. Residents and fellows were all the trainees of a single referral center and were invited by email with in-person reinforcement; specialists were invited individually by email.

**Table 1:** Participants by orthopedic training level, with number invited and response rate

Training level	Invited, n	Participated, n	Response rate	Share of sample
First-year orthopedic resident	7	7	100%	14%
Second-year orthopedic resident	8	8	100%	16%
Third-year orthopedic resident	8	8	100%	16%
Shoulder and elbow fellow	8	8	100%	16%
Shoulder and elbow specialist	51	19	37%	38%
Total	82	50	61%	100%

Angle-specific accuracy

The magnitude and direction of error varied across the elevation arc (Table 2). The mean estimate was nearly unbiased at 90° (89.3°; mean error, -0.7°) and 115° (114.3°; mean error, -0.7°). The largest systematic error occurred at 160°, which was underestimated by a mean of 21.4° (95% CI, -25.7° to -17.2°; Holm-adjusted p < 0.001); this was the only angle at which the mean signed error differed significantly from zero after correction. The 70° target was overestimated by a mean of 5.2° (95% CI, +1.3° to +9.2°), and the 30° target recorded in the sagittal plane was overestimated by a mean of 5.8° (95% CI, -0.5° to +12.2°), but neither finding remained statistically significant after adjustment (adjusted p = 0.063 and p = 0.361, respectively). The mean errors at 30° in the coronal plane, 60°, 90°, and 115° were also not statistically different from zero after correction.

Exact agreement was most frequent at 90°, where 43 of 50 physicians selected the reference value (86%), followed by 30° in the sagittal plane (23/50, 46%), 30° in the coronal plane (19/50, 38%), 60° (15/50, 30%), 70° (11/50, 22%), 160° (4/50, 8%), and 115° (2/50, 4%) (Table 2). The low exact-match rate at 115° despite a mean estimate close to the reference reflects substantial dispersion of individual estimates (standard deviation, 17.5°; range, 50°-150°) and illustrates why exact agreement should not be interpreted as the sole measure of accuracy. The proportion of estimates within ±10° of the reference was 82% at 30° in the coronal plane, 82% at 30° in the sagittal plane, 50% at 60°, 90% at 70°, 96% at 90°, 76% at 115°, and 34% at 160°.

**Table 2:** Angle-specific visual estimation accuracy (n = 50) Signed error was calculated as the estimated angle minus reference angle. Negative values indicate underestimation, and positive values indicate overestimation. One-sample t-tests were two-sided with 49 degrees of freedom, and p-values were adjusted using the Holm method across seven comparisons. The 30° reference angle was assessed in both the coronal and sagittal planes. SD, standard deviation; CI, confidence interval

Reference angle (°)	Plane	Mean estimate (°)	SD (°)	Mean signed error (°)	95% CI of signed error (°)	t (df = 49)	Unadjusted p	Holm-adjusted p	Exact agreement, n (%)
30	Coronal	33.32	21.07	3.32	-2.67 to +9.31	1.11	0.271	0.812	19 (38%)
30	Sagittal	35.82	22.39	5.82	-0.54 to +12.18	1.84	0.072	0.361	23 (46%)
60	Coronal	56.58	15.31	-3.42	-7.77 to +0.93	-1.58	0.121	0.482	15 (30%)
70	Sagittal	75.24	13.91	5.24	+1.29 to +9.19	2.66	0.010	0.063	11 (22%)
90	Coronal	89.30	5.36	-0.70	-2.22 to +0.82	-0.92	0.360	0.812	43 (86%)
115	Sagittal	114.30	17.53	-0.70	-5.68 to +4.28	-0.28	0.779	0.812	2 (4%)
160	Coronal	138.56	14.97	-21.44	-25.69 to -17.19	-10.13	<0.001	<0.001	4 (8%)

Training level and rater effects

Mean absolute error per participant across the seven videos, the signed error at 160°, and the agreement rates by training level are shown in Table 3. Mean absolute error differed across the five training levels (Kruskal-Wallis p = 0.025), but not monotonically with experience: it was lowest among third-year residents (6.6°) and highest among first-year residents (11.5°), with fellows (9.3°) and specialists (10.9°) intermediate. After Holm correction, only the contrast between second- and third-year residents remained significant (p = 0.045). One specialist gave internally inconsistent responses (180° and 170° for the two 30° videos); excluding this participant, the specialist mean was 7.8° (p = 0.015 across levels). Residents, fellows, and specialists as groups did not differ (9.4°, 9.3°, and 10.9°; p = 0.38), and exact agreement did not differ by level (χ² p = 0.80). The mean signed error at 160° was negative at every training level, ranging from -15.0° among fellows to -37.5° among second-year residents.

**Table 3:** Estimation error and agreement by training level Mean absolute error is the mean of the absolute differences between the estimate and the reference across the seven videos for each participant. Kruskal-Wallis test across the five levels for mean absolute error, p = 0.025; χ² test across levels for exact agreement, p = 0.80. The specialist mean absolute error is inflated by one participant with internally inconsistent responses (mean excluding this participant: 7.8°). SD, standard deviation; IQR, interquartile range; CI, confidence interval

Training level	n	Mean absolute error, mean (SD) (°)	Mean absolute error, median (IQR) (°)	Signed error at 160°, mean (95% CI) (°)	Exact agreement, all videos	Within ±10°, all videos
First-year resident	7	11.5 (4.6)	9.3 (8.9-13.4)	-16.6 (-34.6 to +1.5)	14/49 (29%)	31/49 (63%)
Second-year resident	8	10.5 (2.0)	11.4 (9.5-11.9)	-37.5 (-47.2 to -27.8)	16/56 (29%)	38/56 (68%)
Third-year resident	8	6.6 (2.1)	6.9 (5.2-8.0)	-18.8 (-28.2 to -9.3)	20/56 (36%)	47/56 (84%)
Fellow	8	9.3 (5.3)	7.5 (5.8-9.3)	-15.0 (-24.7 to -5.3)	19/56 (34%)	42/56 (75%)
Specialist	19	10.9 (13.9)	7.9 (6.1-9.4)	-20.3 (-26.8 to -13.8)	48/133 (36%)	97/133 (73%)
All	50	10.0 (9.0)	8.3 (6.4-10.4)	-21.4 (-25.7 to -17.2)	117/350 (33%)	255/350 (73%)

In a linear mixed-effects model of absolute error with a random intercept for participant, the effect of the reference angle dominated: absolute error at 160° was 20.9° higher than at 90° (95% CI, 15.9° to 25.8°), whereas fellows and residents differed from specialists by -1.6° (95% CI, -9.2° to +6.0°) and -1.5° (95% CI, -7.0° to +4.1°), respectively; the between-participant standard deviation was 7.8°. The ICC(2,1) for absolute agreement among the 50 raters across the seven videos was 0.85, a value driven mainly by the wide spread of reference angles. The mean within-rater difference between the sagittal and coronal 30° videos was +2.5° (paired t-test p = 0.064; Wilcoxon p = 0.063). Median completion time was 4 min 18 s (interquartile range, 3 min 32 s to 6 min 0 s). No two response sets were identical; three IP addresses were shared by two or three completed responses submitted within minutes of each other, in every case with responses differing on at least three angles. 

## Discussion

This exploratory study demonstrated that visual estimation of active shoulder elevation was strongly angle-dependent. Estimates were, on average, close to the target at 90° and 115°, whereas substantial systematic error was observed at terminal elevation. Elevation at 160° was underestimated by more than 20° on average and was the only angle showing a statistically significant bias after correction. Conversely, 70° and 30° in the sagittal plane were modestly overestimated, by approximately 5° and 6°, respectively, although neither difference reached significance. These findings indicate that the accuracy of visual inspection cannot be assumed to be constant across the shoulder elevation arc.

One possible explanation for the near-zero mean error at 90° is that the horizontal position of the arm provides a familiar external visual reference, which may reduce perceptual uncertainty. This is speculation. The study was not designed to test cognitive or perceptual mechanisms, and no such mechanism is demonstrated by these data. At the group mean level, the estimate at 115° was almost unbiased, lying only 0.7° below the reference. This should not be read as accuracy at the individual level: individual estimates were widely dispersed (SD, 17.5°), and exact agreement occurred in only 4% of ratings. Mean bias and dispersion are therefore more informative than exact agreement alone when the response tool permits continuous values.

The marked underestimation at 160° may reflect the difficulty of judging terminal elevation from a two-dimensional recording. Several factors could contribute and were not controlled: the framing of the video; the height and distance of the camera; lens distortion at the periphery of the frame; the size and orientation of the screen on which each participant viewed the clip; the relationship between the humerus and the thorax as rendered in two dimensions; and the scapulothoracic contribution to elevation, which was not measured. Intermediate angles may also lack obvious environmental reference lines, increasing perceptual error. Previous studies have similarly shown that the reliability and error of shoulder ROM assessment vary by movement and measurement technique [5,8-10,18].

Because the 30° position was recorded in both the coronal and sagittal planes, the two videos could be compared within raters. The mean signed error was +3.3° in the coronal plane and +5.8° in the sagittal plane, and the mean within-rater difference (sagittal minus coronal) was +2.5° (paired t-test p = 0.064; Wilcoxon signed-rank p = 0.063). This post hoc comparison of a single reference angle in a single model does not demonstrate equivalence between planes and should not be read as evidence for or against an effect of plane on estimation accuracy; the study was not designed to compare planes.

Our findings are consistent with primary evidence on visual estimation of shoulder ROM. Terwee et al. assessed 201 patients with shoulder complaints and found that the lowest interobserver agreement was for visual estimation of active and passive elevation, with limits of agreement exceeding 40° [6]. Hayes et al. compared five assessment methods in symptomatic patients and reported standard errors of measurement between 14° and 25° across raters, with visual estimation performing no better than the other methods [19]. Hwang et al. found generally high interobserver reliability among shoulder surgeons but limited accuracy compared with digital goniometry, with errors that could exceed clinically important thresholds [14]. Our own data illustrate the same distinction: the ICC among raters was 0.85, yet only one in three estimates at 160° fell within ±10° of the reference, and every training level underestimated it. Agreement among clinicians does not establish validity: multiple observers can provide similar estimates while sharing the same directional bias.

Participants spanned the full range of orthopedic training, and this heterogeneity was intentional. Mean absolute error differed across the five training levels but not in the direction that increasing experience would predict: third-year residents were the most accurate, specialists were not more accurate than residents, and the group effects in the mixed-effects model were small relative to the effect of the reference angle. This is consistent with the cadaveric study by Russo et al., in which level and type of examiner experience produced statistically detectable but not clinically meaningful differences in range-of-motion measurement [16]. Whatever the mechanism, the angle-dependent bias observed here was shared across training levels rather than confined to less experienced observers.

Validated digital tools offer practical alternatives, but validity is a property of a specific device, application, and protocol rather than of digital measurement in general. Smartphone inclinometers and dedicated applications have demonstrated acceptable to excellent validity and reliability in several settings [10-12,20], while a systematic review of clinically accessible applications found that relative measures of agreement were consistently stronger than absolute ones, meaning that a given application may track change well while differing systematically from a criterion measure [12]. Clinicians should therefore select a tool with published evidence for the joint and movement of interest, apply a standardized protocol, and avoid switching instruments between assessments of the same patient. Their use may be particularly valuable in telemedicine, multicenter research, postoperative follow-up, and any context in which relatively small ROM changes influence interpretation.

This study has strengths. All observers assessed the same video stimuli, reference angles were checked using two measurement approaches, the sample spanned physicians from the first year of residency through subspecialty practice, and the participant-level dataset is available. These features support internal comparability across angles. They do not establish external generalizability: participants were trainees and fellows from a single high-complexity referral center, together with specialists invited by email, and the sample does not represent general orthopedic practice, primary care, physiotherapy, or telemedicine users.

Several limitations must be acknowledged. No a priori sample-size calculation was performed, and the analyses beyond the angle-specific signed error were exploratory and not prespecified. The seven videos were presented in the same fixed order to every participant, so learning, fatigue, and anchoring effects cannot be separated from the effect of the reference angle; in particular, the 160° video was the first item presented, and an anchoring or warm-up effect cannot be distinguished from a genuine perceptual difficulty at terminal elevation. Exact agreement for the 30° coronal video was mis-tabulated in the original analysis and has been corrected in this version.

Participants spanned a wide range of clinical experience. This was intentional and directed at reflecting the range of clinicians who record visual estimates in practice. Mean absolute error differed across the five training levels but not monotonically with experience, and specialists were not more accurate than residents; with seven to eight participants per resident and fellow category, however, these comparisons have limited power and should be regarded as exploratory. The questionnaire recorded level of training only; years in practice for specialists, time since fellowship, and shoulder case volume were not captured, and future studies should record them.

The reference procedure was not validated against three-dimensional motion analysis, which remains the criterion standard for angular measurement. Every position was accepted only after the goniometric and phyphox readings agreed within a prespecified 5° tolerance, but the individual paired readings were not documented in writing at the time of filming, so this agreement cannot be independently verified from a record. The tolerance is also proportionally larger at low target angles; phyphox has not been formally validated as a clinical goniometric instrument; and trunk position was monitored visually rather than instrumented. Residual error in the reference angles themselves therefore cannot be excluded, and future studies should record and publish the paired readings for every reference position.

Only seven videos, one movement domain, and one healthy model were used; the scapular plane, which is frequently used in clinical practice, was not assessed, and only two planes were sampled, with a single angle common to both. Patients with shoulder disorders may compensate for restricted glenohumeral elevation through trunk inclination or other movements, potentially making visual estimation more difficult than in this setting. Screen size, viewing device, ambient conditions, and the ability to replay clips were not recorded or controlled.

Recruitment used a convenience sample from a single referral center plus invited specialists. Whereas all residents and fellows participated, only 37% of invited specialists did, so the specialist subgroup is self-selected and may not represent specialists in general. The anonymous design means that respondent uniqueness, although supported by the IP-based audit described in the Methods, cannot be verified with certainty. No second assessment was performed, so intraobserver reliability could not be evaluated. Exact agreement is highly sensitive to slider behavior and is reported as a secondary descriptive endpoint only.

Future studies should use a larger number of standardized videos, randomize presentation order, record the viewing device, include symptomatic patients, record years of practice and case volume, and repeat the assessment after an interval to estimate intraobserver reliability.

## Conclusions

Within this video-based experimental setting, visual estimation of active shoulder elevation demonstrated angle-dependent accuracy. Estimates were closest to the reference at 90° and, at the group mean level, at 115°, whereas 160° was systematically underestimated by more than 20° at every level of training; 70° and 30° in the sagittal plane were modestly overestimated. A visually estimated ROM value may therefore be misleading even when the examination appears straightforward. The study did not evaluate screening performance, functional categorization, symptomatic patients, or in-person examination, and no claim about those settings is made. Where precise documentation, comparison over time, outcome-score calculation, or research measurement is required, a standardized universal goniometer, digital inclinometer, or validated smartphone-based tool applied under a reproducible protocol should be preferred over visual estimation.

## Appendices

Appendix 1

**Table 4:** Participant-level responses (50 rows: training level, seven estimates, start and completion timestamps; IP addresses removed)

Supplementary Data - Participant Level (n = 50)										
Participant	Start utc	Completion_utc	Training Level	Video 1	Video 2	Video 3	Video 4	Video 5	Video 6	Video 7
1	2022-11-23 08:51:21	2022-11-23 08:55:50	Specialist	150.0	90.0	30.0	19.0	80.0	45.0	100.0
2	2022-11-29 19:05:09	2022-11-29 19:07:14	Specialist	150.0	90.0	30.0	30.0	80.0	45.0	120.0
3	2022-11-30 06:07:34	2022-11-30 06:11:51	R2	140.0	90.0	30.0	30.0	80.0	70.0	110.0
4	2022-12-06 10:32:04	2022-12-06 10:36:38	Fellow	140.0	90.0	30.0	30.0	40.0	30.0	50.0
5	2022-12-06 10:37:12	2022-12-06 10:41:03	Fellow	130.0	90.0	30.0	20.0	80.0	60.0	120.0
6	2022-12-06 10:39:58	2022-12-06 12:07:44	Specialist	140.0	90.0	30.0	20.0	70.0	45.0	120.0
7	2022-12-06 10:43:21	2022-12-06 10:47:16	Fellow	150.0	90.0	30.0	30.0	80.0	45.0	110.0
8	2022-12-06 10:52:03	2022-12-06 11:12:56	Specialist	135.0	90.0	30.0	30.0	70.0	45.0	150.0
9	2022-12-06 10:52:44	2022-12-06 10:58:43	Specialist	150.0	90.0	30.0	33.0	70.0	45.0	110.0
10	2022-12-06 12:41:38	2022-12-06 12:44:57	Specialist	160.0	90.0	30.0	30.0	60.0	45.0	120.0
11	2022-12-06 13:02:38	2022-12-06 13:05:20	R1	130.0	90.0	30.0	30.0	61.0	80.0	110.0
12	2022-12-06 13:13:30	2022-12-06 13:18:31	Specialist	140.0	90.0	29.0	29.0	79.0	45.0	120.0
13	2022-12-06 17:08:33	2022-12-06 17:17:54	R3	120.0	90.0	30.0	30.0	70.0	45.0	120.0
14	2022-12-06 17:12:43	2022-12-06 17:16:11	R3	130.0	90.0	30.0	30.0	75.0	45.0	110.0
15	2022-12-06 17:31:44	2022-12-06 17:39:27	Specialist	160.0	90.0	29.0	30.0	70.0	50.0	120.0
16	2022-12-06 17:47:15	2022-12-06 17:50:27	R3	140.0	90.0	45.0	31.0	80.0	45.0	111.0
17	2022-12-06 17:50:32	2022-12-06 17:54:51	Fellow	145.0	90.0	40.0	30.0	80.0	60.0	120.0
18	2022-12-06 18:14:56	2022-12-06 18:20:58	R3	140.0	90.0	40.0	40.0	75.0	70.0	115.0
19	2022-12-06 18:20:53	2022-12-06 18:24:27	R3	150.0	90.0	30.0	19.0	80.0	60.0	105.0
20	2022-12-06 18:24:02	2022-12-06 18:27:54	Specialist	135.0	90.0	30.0	30.0	70.0	45.0	120.0
21	2022-12-06 18:40:37	2022-12-06 18:44:08	R3	150.0	90.0	40.0	35.0	80.0	60.0	120.0
22	2022-12-07 05:29:22	2022-12-07 05:35:23	Fellow	140.0	90.0	29.0	40.0	79.0	60.0	100.0
23	2022-12-07 10:16:38	2022-12-07 10:21:29	Specialist	144.0	88.0	35.0	38.0	75.0	69.0	111.0
24	2022-12-07 10:24:23	2022-12-07 10:28:36	Specialist	120.0	90.0	30.0	30.0	60.0	60.0	120.0
25	2022-12-07 18:30:45	2022-12-07 18:34:35	Specialist	120.0	90.0	40.0	30.0	80.0	60.0	110.0
26	2022-12-08 09:15:57	2022-12-08 09:21:11	Specialist	129.0	90.0	30.0	30.0	75.0	45.0	120.0
27	2022-12-08 18:54:48	2022-12-08 18:58:12	R2	140.0	90.0	45.0	45.0	70.0	70.0	120.0
28	2022-12-12 13:23:57	2022-12-12 13:32:22	Fellow	140.0	90.0	40.0	26.0	76.0	61.0	115.0
29	2022-12-12 13:34:21	2022-12-12 13:38:26	R3	150.0	90.0	31.0	30.0	80.0	60.0	110.0
30	2022-12-12 13:59:51	2022-12-12 14:06:34	R2	110.0	90.0	30.0	31.0	75.0	45.0	102.0
31	2022-12-12 14:05:18	2022-12-12 14:09:37	Specialist	150.0	90.0	19.0	45.0	70.0	60.0	140.0
32	2022-12-12 16:49:40	2022-12-12 16:54:47	Specialist	150.0	90.0	30.0	40.0	80.0	60.0	110.0
33	2022-12-12 16:51:25	2022-12-12 16:54:54	Specialist	120.0	90.0	40.0	29.0	80.0	60.0	120.0
34	2022-12-12 16:53:11	2022-12-12 17:02:08	Specialist	120.0	70.0	70.0	45.0	70.0	71.0	121.0
35	2022-12-12 17:02:16	2022-12-12 17:06:53	Specialist	150.0	100.0	180.0	170.0	150.0	100.0	150.0
36	2022-12-12 17:02:33	2022-12-12 17:15:18	Specialist	131.0	90.0	35.0	35.0	83.0	51.0	110.0
37	2022-12-12 20:09:50	2022-12-12 20:12:59	R1	130.0	60.0	29.0	30.0	80.0	75.0	110.0
38	2022-12-16 14:18:11	2022-12-16 14:21:46	R1	119.0	95.0	30.0	30.0	60.0	40.0	50.0
39	2022-12-16 14:25:08	2022-12-16 14:31:06	R2	120.0	90.0	41.0	29.0	70.0	50.0	120.0
40	2022-12-16 19:19:00	2022-12-16 19:23:05	R2	120.0	90.0	45.0	20.0	79.0	60.0	110.0
41	2022-12-16 19:22:12	2022-12-16 19:25:47	R2	120.0	90.0	31.0	40.0	81.0	45.0	109.0
42	2022-12-19 05:04:49	2022-12-19 05:08:11	R2	120.0	90.0	45.0	36.0	80.0	60.0	100.0
43	2022-12-28 09:28:40	2022-12-28 09:30:45	R1	160.0	90.0	25.0	20.0	80.0	45.0	110.0
44	2023-01-02 08:47:22	2023-01-02 08:49:22	R1	160.0	90.0	20.0	15.0	80.0	45.0	130.0
45	2023-01-02 08:49:52	2023-01-02 08:51:25	R1	170.0	90.0	30.0	15.0	80.0	50.0	130.0
46	2023-01-02 12:07:33	2023-01-02 12:11:10	R3	150.0	90.0	30.0	31.0	80.0	62.0	111.0
47	2023-01-02 13:48:10	2023-01-02 13:58:03	R2	110.0	90.0	29.0	20.0	79.0	60.0	105.0
48	2023-01-02 19:31:50	2023-01-02 19:41:14	R1	135.0	91.0	30.0	45.0	50.0	60.0	150.0
49	2023-01-02 19:48:08	2023-01-02 19:53:25	Fellow	170.0	91.0	34.0	35.0	60.0	120.0	120.0
50	2023-01-02 20:02:57	2023-01-03 14:44:28	Fellow	145.0	90.0	15.0	30.0	70.0	45.0	120.0
